# Low-Velocity Impact Resistance of Al/Gf/PP Laminates with Different Interface Performance

**DOI:** 10.3390/polym13244416

**Published:** 2021-12-16

**Authors:** Yanyan Lin, Huaguan Li, Zhongwei Zhang, Jie Tao

**Affiliations:** 1 College of Material Science and Technology, Nanjing University of Aeronautics and Astronautics, Nanjing 211106, China; linyanyan@nuaa.edu.cn; 2 Jiangsu Key Laboratory of Advanced Structural Materials and Application Technology, Nanjing Institute of Technology, Nanjing 211167, China; 3 State Key Laboratory of Explosion & Impact and Disaster Prevention & Mitigation, Army Engineering University of PLA, Nanjing 210007, China; zhangzhongwei.cn@gmail.com; 4 Jiangsu Collaborative Innovation Center for Advanced Inorganic Function Composites, Nanjing 210016, China

**Keywords:** Al/Gf/PP laminates, low-velocity impact, interface performance, plasma surface treatment, fracture toughness

## Abstract

The weak interface performance between metal and composite (IPMC) makes the composite materials susceptible to impact load. Aluminum/glass fiber/polypropylene (Al/Gf/PP) laminates were manufactured with the aluminum alloy sheets modified by nitrogen plasma surface treatment and the phosphoric acid anodizing method, respectively. FEM models of Al/Gf/PP laminates under low-velocity impact were established in ABAQUS/Explicit based on the generated data including the model I and II interlaminar fracture toughness. Low-velocity impact tests were performed to investigate the impact resistance of Al/Gf/PP laminates including load traces, failure mechanism, and energy absorption. The results showed that delamination was the main failure mode of two kinds of laminates under the impact energy of 20 J and 30 J. When the impact energy was between 40 J and 50 J, there were metal cracks on the rear surface of the plasma pretreated specimens, which possessed higher energy absorption and impact resistance, although the integrity of the laminates could not be preserved. Since the residual compressive stress was generated during the cooling process, the laminates were more susceptible to stretching rather than delamination. For impact energy (60 J) causing the through-the-thickness crack of two kinds of laminates, plasma pretreated specimens exhibited higher SEA values close to 9 Jm^2^/kg due to better IPMC. Combined with the FEM simulation results, the interface played a role in stress transmission and specimens with better IPMC enabled the laminates to absorb more energy.

## 1. Introduction

Fiber metal laminates (FMLs) have been designed to improve the fatigue life and damage tolerance of the metal in aerospace applications [1,2,3]. Glass fiber reinforced aluminum laminates (GLARE) are one of the most representative and have been successfully applied in A380 due to their contribution to reducing weight and saving costs [4,5]. The consolidation of traditional thermoset-based FMLs requires a simultaneous combination of high temperature, high pressure, and long process cycle time. Nevertheless, thermoplastic-based (TP-based) FMLs have the advantage of better toughness, high production efficiency, excellent recyclability, and thermal deformation [6,7]. With the continuous development of the public transportation industry, TP-based FMLs have attracted attention from many experts and scholars. A glass fiber reinforced thermoplastic layer alternatively laminated with an aluminum alloy sheet has great application prospects, mainly in new energy vehicles and rail transit industries. These high-performance composites with the benefit of superior safety and performance–price ratio could be used in the manufacture of energy absorption boxes and battery boxes for new energy vehicles as well as the skin structure of high-speed trains [8,9,10].

FMLs present excellent energy absorption capacity and relatively low damage, especially after low-velocity impact since the adhesive is combined with the elastoplasticity of the metal and the stiffness of the fiber reinforced resin matrix composites [11]. Regarding the research of impact response of FMLs, academics have mainly focused on the effects of metal type, fiber type, matrix type, metal/composite volume fraction, and lay-up configuration on impact damage [12,13,14]. Fan et al. [15] studied the low-velocity impact tests on GLARE and found that the perforation resistance of all test specimens increased with the target thickness, plate diameter, and impactor radius. Furthermore, significant areas of delamination failure outside the impacted area in the cross-sections and the surface treatment method of the aluminum alloy sheet have not been mentioned. An analogous failure was found in [16], in which the specimen was treated by a standard anodizing method. It was noted that the delamination was the primary failure mode in FMLs, and the interlaminar shear properties played an essential role [17]. In other words, the weak interface performance between metal and composite (IPMC) makes the composite materials susceptible to impact load [18]. It makes sense to investigate the mechanism of IPMC on the dynamic response of FMLs under the safety design for practical applications. Serious damage could be induced even when the structures are impacted with low-velocity energy, causing a significant reduction in compressive strength [19]. Therefore, great efforts should be given to the design of the skin structure of higher-speed trains to withstand impacts from various objects (large ice cubes, birds, and small rocks, etc.) during manufacturing, maintenance, and service procedures.

Glass fiber reinforced polypropylene (GFPP) is attractive for manufacturing TP-based FMLs due to its low cost and high performance. Nevertheless, component PP has poor adhesion to other materials due to a high cooling contraction percentage [20]. Therefore, surface treatment of the metal sheet is crucial in the preparation progress of FMLs. The physical and chemical properties of the metal sheet surface can significantly influence the IPMC of FMLs. Zhu et al. [21] modified the surface of aluminum alloy AA6061 with treatments (sanding, anodizing, silane coupling agent) to improve IPMC. They found that the effect with a silane coupling agent was similar to that of phosphoric acid anodizing (PAA), which was better than sanding. Mehr et al. [22] investigated the effect of different surface treatments on the flexural and impact behavior of FMLs including forest product laboratory etching (FPL), sulfuric acid anodizing (SAA), and the combination of sandblasting with FPL and SAA. The results demonstrated that the adhesion between the metal and composite was more critical in impact testing than in flexural testing. In addition, the surface treatment combined with sandblasting and SAA was the best candidate for manufacturing FMLs. However, although chemical methods can roughen the metal surface to improve the IPMC, the resulting chemical waste liquid is harmful to the environment. In recent years, plasma surface treatment has increasingly been used for surface modification of metal and composites for the characteristics of high efficiency, low-cost, and being pollution-free [23]. We have considered the effect of various parameters for plasma surface treatment on the static mechanical properties of aluminum/glass fiber/polypropylene (Al/Gf/PP) laminates. Results indicated that 10 min-nitrogen plasma treatments for aluminum alloy sheets could make the mechanical performance of Al/Gf/PP laminates better than that of PAA due to the mechanical and chemical bonding with GFPP [24]. However, the effects of IPMC formed by these two methods on the low-velocity impact resistance of Al/Gf/PP laminates are still unknown and require further analysis.

In addition to the experimental methods, finite element method (FEM) simulation plays an important part in the performance analysis of engineering structures and materials. Gohari et al. [25,26] proposed analytical solutions for obtaining static deformation and optimal shape control of composite hybrid plates and beams under thermo-electro-mechanical loads through FEM analysis. Moreover, an analytical solution was established for elastic flexure of thick multi-layered composite hybrid plates resting on a Winkler elastic foundation when the plate was in the air or submerged in water, and its accuracy was verified by the FEM simulation. Iqbal et al. [27] studied the drop impact resistance of prestressed concrete plates and performed the FEM method using the Holmquist–Johnson–Cook constitutive model for concrete. Katsamakas et al. [28] simulated the failure modes and force–displacement of shear deficient R/C beams strengthened with steel reinforced grout through embedded truss elements. The FEM-based numerical analysis predicted the observed crack pattern and failure modes accurately, whereas deviation in terms of load and deflection was, on average, less than 1% and 10%, respectively.

This work aims to investigate the low-velocity resistance of Al/Gf/PP laminates with different IPMC through numerical and experimental methods. The aluminum alloy sheets for manufacturing these TP-based FMLs were modified by nitrogen plasma surface treatment and the phosphoric acid anodizing method, respectively. Interlaminar fracture toughness of Models I and II was tested by a single cantilever beam and end notch bending methods. Low-velocity impact tests were conducted to investigate the impact resistance of Al/Gf/PP laminates including load traces, failure mechanism, and energy absorption.

## 2. Materials and Methods

### 2.1. Materials

Glass fiber reinforced polypropylene prepregs (GFPP, Shanghai Genius Advanced Materials Co. Ltd. Shanghai, China) were used to prepare the composite part of the Al/Gf/PP laminates. The essential performance is presented in Table 1. The 6061-T6 aluminum alloy sheet (thickness 0.5 mm, Southwest Aluminum Group Co. Ltd. Chongqing, China) was machined to the size of 300 mm × 300 mm before the metal surface treatments. An industrial film (thickness 0.04 mm, L461A-04, Shanghai Hehe Hotmelt Adhesives Co. Ltd. Shanghai, China) with the main content of polypropylene was placed between the metal and prepregs to enhance the interfacial adhesion.

### 2.2. Surface Treatments

The surface treatment method of the aluminum alloy sheet used in this work was nitrogen plasma treatment for 10 min [24] and the phosphoric acid anodizing method [29].

### 2.3. Specimen Preparation

FMLs are a layered composite material with symmetrical structures to ensure the structural stability. The Al/Gf/PP laminates with dimensions of 300 mm × 300 mm × 2.7 mm were stacked with a sequence of [A/C/F0/F90/C/A/C/F90/F0/C/A], in which A, C, F0, and F90 respectively refer to aluminum alloy layer, cohesive layer and GFPP layer with orientations of 0° and 90° (0° is the rolling direction of aluminum alloy sheet, and the direction perpendicular to it is 90°). The specimen wrapped with the release cloth was placed horizontally in the vulcanizer equipment (XLB-D 0.1 MN, 400 × 400 × 1). The curing temperature was 180 °C for 5 min and the pressure of 0.4 MPa was applied before the specimen was cooled to 80 °C. Finally, the specimen was taken out carefully and cooled in the air at room temperature.

### 2.4. Fracture Toughness Tests

**Figure 1:**
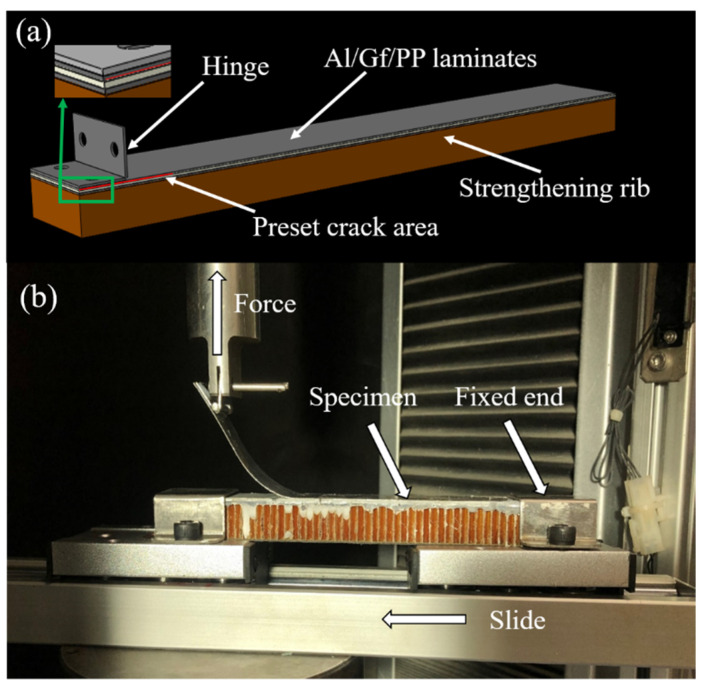
Mode I interlaminar fracture toughness: (**a**) the specimen of Al/Gf/PP laminates, (**b**) the single cantilever beam (SCB) method.

The mode I interlaminar fracture toughness of resin matrix composites is generally referred to in ASTM D5528-13 by the double cantilever beam (DCB) test. However, the accuracy of the results for FMLs is problematic due to its asymmetrical structure after prefabricated cracks [30]. Therefore, the single cantilever beam (SCB) method was used and the specimen is shown in Figure 1. The specimen consisted of a 2/1 structure, which is two layers of aluminum alloy sheets with a layer of GFPP prepregs in between. The thickness direction of the specimen was coated with white liquid for better observation of crack propagation. The size of the specimen was 180 mm × 25 mm × 1.6 mm. The preset crack length was 50 mm and the hinge size was 15 mm × 25 mm. Experiments were conducted at the rate of 1 mm/min on the testing machine (AGS-X10KN, Shimadzu, Japan). When the crack propagation reached 20 mm, data started to be recorded until the crack length reached 100 mm.

The formula for mode I interlaminar fracture toughness is as follows:

(1) $$G_{IC}=\frac{3P^{2}ea^{2}}{2b}$$

where *P* is the peak force when each segment of the crack grows; *a* is the length of each segment of the crack; and *b* is the width of the specimen. In addition, *e* is the slope of the function curve drawn by *a*^3^ and flexibility (*C*, the ratio of displacement to peak force).

**Figure 2:**
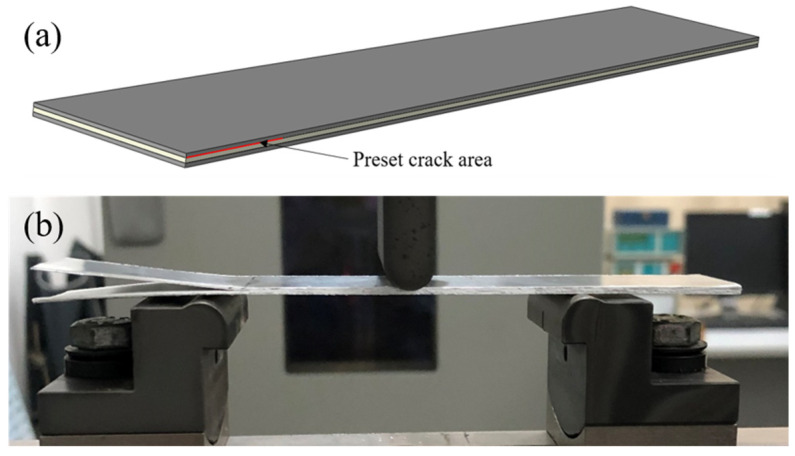
Mode II interlaminar fracture toughness: (**a**) the specimen of Al/Gf/PP laminates, (**b**) the end notch bending test.

The mode II interlaminar fracture toughness was tested by the end notch bending (ENB) method according to the standard ASTM D7905. The size was 140 mm × 25 mm, and the preset crack length between the aluminum alloy and GFPP layer was 40 mm (Figure 2a). As shown in Figure 2b, the radius of the indenter and the support were 5 mm, and the span was 70 mm. The test rate was 1 mm/min.

The calculation formula for mode II interlaminar fracture toughness is as follows:

(2) $$G_{IIC}=\frac{9P\delta a^{2}}{2W(L^{3}/4+3a^{2})}\times 10^{3}$$

where *P* is the maximum load after crack propagation; *δ* is the deflection of the specimen under load; *a* is the effective crack length; *W* is the width of the specimen; and *L* is the span.

### 2.5. Low-Velocity Impact Testing

The specimens with the size 150 mm × 100 mm (ASTM D7136/D7136M-15) were prepared by water cutting, where the rolling direction of the aluminum alloy sheet was parallel to the long side. Low-velocity impact tests were performed by a drop-weight impact tester (Ceast/Instron 9350, Instron, Norfolk County, MA, US) and repeated three times each under the same conditions. During the test, the specimen was sandwiched between two steel plates with a diameter of 50 mm in the center, and four sides were fixed on the bottom support. A hemisphere impactor with a diameter of 16 mm and a mass of 5.41 kg was used. Specimens pretreated with different surface treatments were impacted with the same energy level. The velocity of the impactor was maintained between 2.72 m/s and 4.76 m/s. Thee range of impact energy was obtained by adjusting the drop height, varying from 20 J to 60 J. The impact energy was selected to achieve the through-the-thickness (TTT) crack, which occurred on both sides of the laminates [31]. Some of the impacted specimens were machined into two halves through the impacted center by the bench saw. Post-mortem micro-fractography was taken to record the detailed damage patterns by the impact.

### 2.6. Finite Element Models

**Figure 3:**
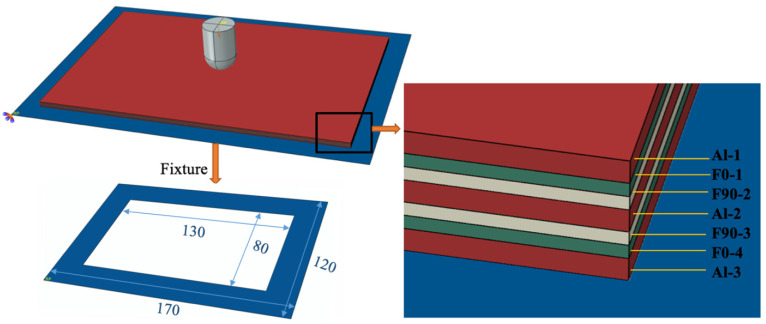
The numerical model of low-velocity impact for Al/Gf/PP laminates.

The FEM software ABAQUS/Explicit (Dassault, Paris, France) was used for low-velocity impact on Al/Gf/PP laminates. The geometric size of the model was considered using actual dimensions of the experimental specimens including the impactor and Al/Gf/PP laminates. As shown in Figure 3, the impactor was placed close to the top of the laminates and only retained the freedom degree of the impact direction, and the initial speed was set in a predefined field to obtain the low-velocity impact energy. The specimen was placed on a discrete rigid fixture, which was constrained to all degrees of freedom. Comprehensively considering the computational time and accuracy, the general mesh size was chosen to be 2 mm while the average mesh size at the middle part (100 mm × 80 mm) of the structure was carefully refined to be 1.5 mm [32]. The number of element nodes and elements were 28,412 and 14,872, respectively. Moreover, the numerical simulation is more of a qualitative than quantitative character due to the lack of detailed numerical error analysis, which may require the application of non-commercial software for parametric convergence studies and/or adaptive analysis, respectively.

The aluminum alloy sheet and glass fiber reinforced polypropylene layer was created using 8-node 3D reduced elements (C3D8R). The impactor and the fixture were created separately using a 4-node 3D rigid triangular facet element (R3D4). The mechanical behavior of the aluminum alloy sheet was simulated by the Johnson–Cook structural model and relevant constants of Al6061-T6 are shown in Table 2 [33,34,35].

The model is expressed as follows:

(3) $$\sigma =[A+B\varepsilon_{p}^{n}][1+C\ln\frac{\dot{\varepsilon }}{{\dot{\varepsilon }}_{0}}][1-T^{*m}]$$

where *A*, *B*, and *C* are the material parameters; $\varepsilon_{p}$ is the equivalent plastic strain; *n* and *m* are the material constant; $\frac{\dot{\varepsilon }}{{\dot{\varepsilon }}_{0}}$ is the dimensionless plastic strain rate for ${\dot{\varepsilon }}_{0}=1$ s^−1^ and T* is the homologous temperature maintained at zero because the temperature variation was not a consideration in this work. To simulate the ductile damage of the aluminum alloy sheet, the Johnson–Cook damage criteria was used to determine the equivalent plastic strain $\varepsilon_{D}^{pl}$ at the beginning of damage:

(4) $$\varepsilon_{D}^{pl}=(d_{1}+d_{2}e^{-d_{3}\eta })(1+d_{4}\ln\frac{{\dot{e}}^{pl}}{{\dot{e}}_{0}})$$

where η is the stress triaxiality parameter and *d*_1_*–d*_4_ are material parameters [36].

The damage in the glass fiber reinforced polypropylene layer was judged by the 3D Hashin criterion with the use of the VUMAT subroutine and the mechanical properties were obtained experimentally and are listed in Table 3 [37,38].

## 3. Results and Discussion

### 3.1. Fracture Toughness Properties

**Figure 4:**
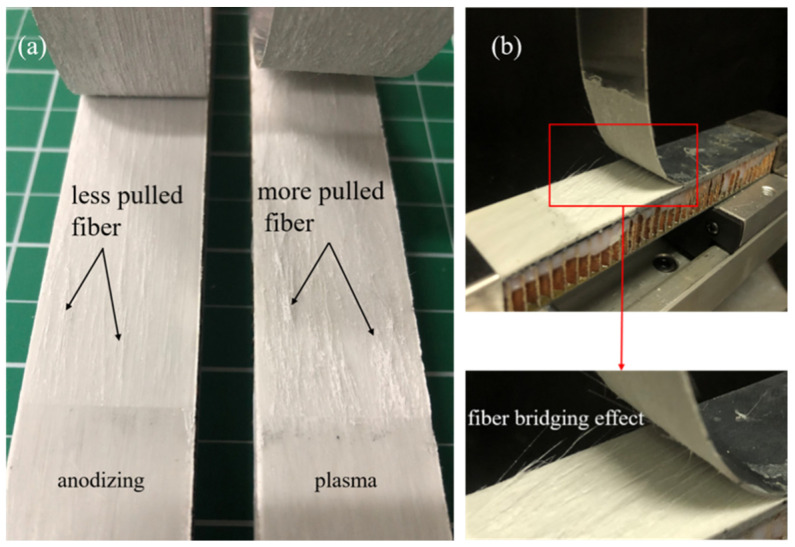
The typical failure modes of mode I interlaminar fracture toughness: (**a**) failure modes of laminates with different IPMC, (**b**) fiber bridging effect of laminates by plasma surface treatment.

**Figure 5:**
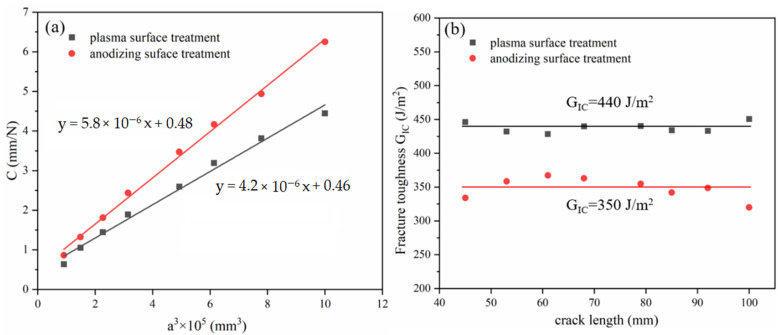
Analysis of the SCB test results: (**a**) flexibility fitting curve, (**b**) R curve.

**Figure 6:**
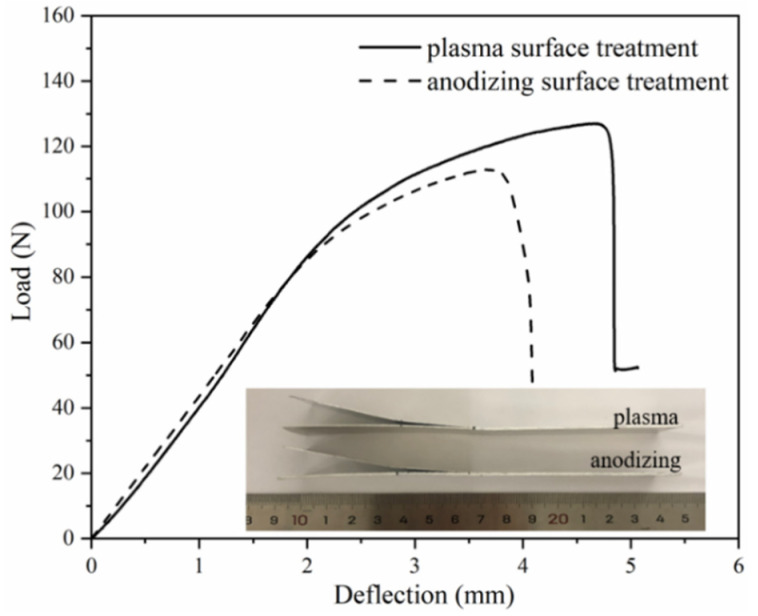
Typical load–deflection curve and failure modes of the ENB test results.

The typical failure modes of mode I interlaminar fracture toughness of laminates with different IPMC are displayed in Figure 4. Laminates with the aluminum alloy layer by plasma pretreatment had more fiber pullouts during the SCB test (Figure 4a). In Figure 4b, PP resin and glass fiber remained on the stripped aluminum alloy sheet, and a large number of fibers in the GFPP layer were pulled out, which played a fiber bridging effect. Figure 5 shows the calculation process and results of mode I interlaminar fracture toughness, which was 0.440 kJ/m^2^ (plasma) and 0.350 kJ/m^2^ (anodizing), respectively. Figure 6 displays the typical load–deflection curve and failure modes of mode II interlaminar fracture toughness. The effective crack length of the specimen was 26 mm, and the mode II interlaminar fracture toughness was 0.500 kJ/m^2^ (plasma) and 0.247 kJ/m^2^ (anodizing), respectively. Parameters of IPMC of Al/Gf/PP laminates with different surface treatments are listed in Table 4, among which the interfacial strength refers to the literature [24].

### 3.2. Force–Time Curves

**Figure 7:**
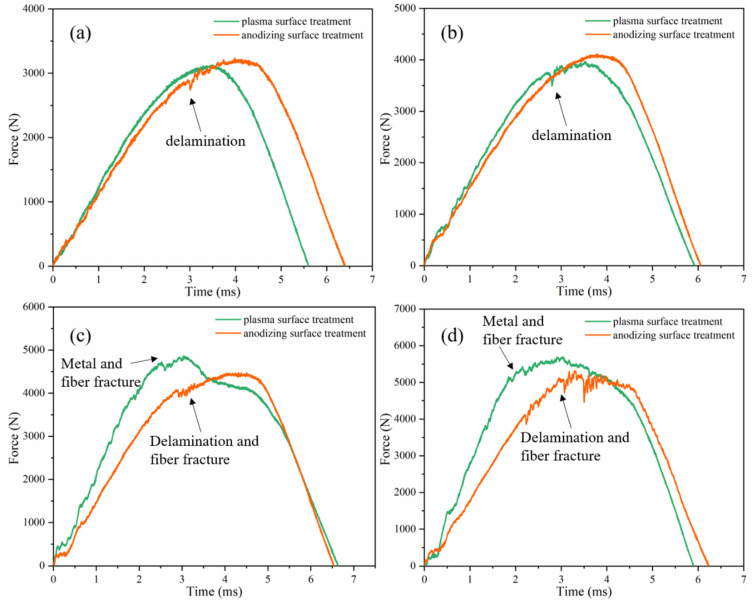
Load traces of Al/Gf/PP laminates subjected to low-velocity impact with different energies: (**a**) 20 J, (**b**) 30 J, (**c**) 40 J, (**d**) 50 J.

Figure 7 shows the force–time curve of the Al/Gf/PP laminates subjected to different low-velocity impact energy. When the impact energy was 20 J and 30 J, the ascending section of the force was not smooth. Some fluctuations could be detected near the area of the peak impact force in Figure 7a,b, which characterized the delamination between thee metal and composite interface. Then, the descending section of the load was relatively smooth. Under the impact energy of 40 J and 50 J (Figure 7c,d), the slope of the curve of the specimen after plasma surface treatment was greater, and the maximum impact load was higher than that after anodization. The plasma surface treatment of aluminum alloy improved the stiffness of the Al/Gf/PP laminates and had a higher impact resistance at this energy level, which was manifested by the fracture failure of metal and fiber. However, the anodized specimen only experienced delamination and fiber fracture failure under the impact of this energy level.

**Figure 8:**
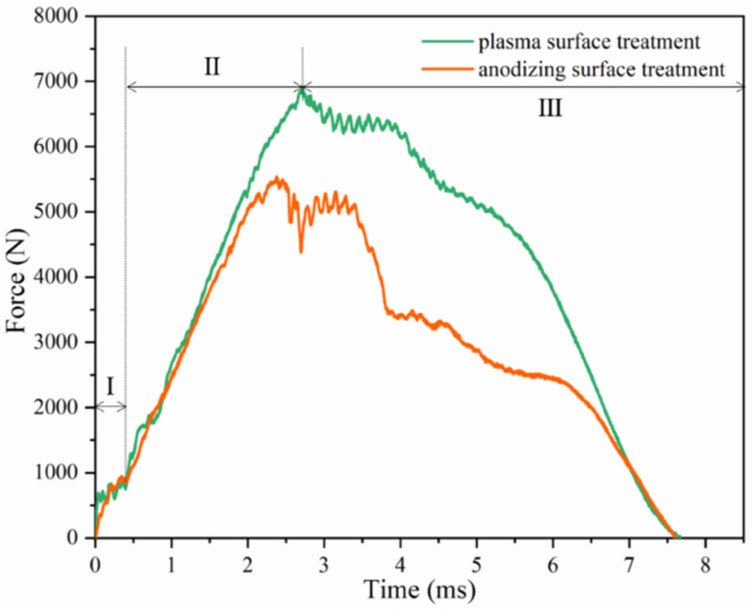
Load traces of Al/Gf/PP laminates subjected to low-velocity impact energy of 60 J.

When the laminates were subjected to the impact energy of 60 J, the corresponding curves were different from that of lower energy (Figure 8), which could be divided into three stages. In the first stage, there was a fluctuation of 0.5 ms, which was caused by the stable process of the system composed of the impactor and the specimen [40]. In the second stage, the specimen after plasma surface treatment and after anodization reached the maximum at 2.9 ms and 2.5 ms, respectively. When the impact force reached its peak value, the back surface of the laminates was broken and entered the third stage. At this stage, the load decreased with the occurrence of multiple failure modes until the specimens were broken down and fully penetrated.

**Figure 9:**
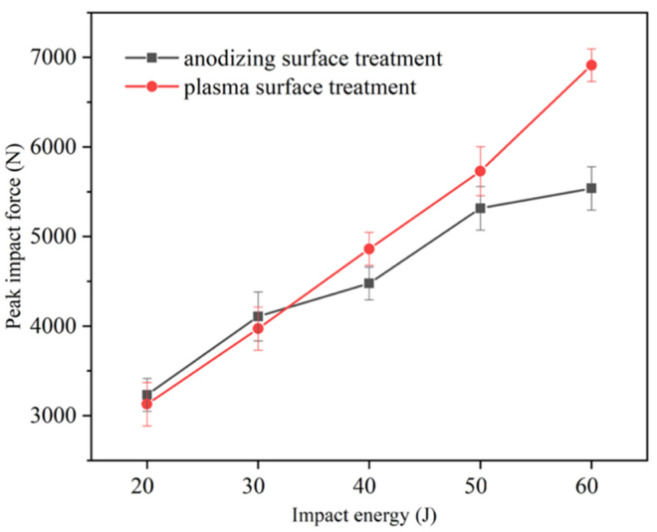
Comparison of the peak impact force between two kinds of Al/Gf/PP laminates subjected to low-velocity impact energy (20–60 J) with the aluminum alloy sheet modified by different surface treatments.

The comparison of the peak impact force of laminates with different aluminum surface treatments is presented in Figure 9. The impact force rises with the increase in impact energy. When the impact energy was below 30 J, the peak impact force of anodizing pretreated specimens was 3.4% higher than that of plasma pretreated specimens, which was almost equal within the error range. In other words, the effect of IPMC at this level of the impact energy was not obvious, which has also been discovered in titanium FMLs [41]. When the impact energy was 40 J and 50 J, the peak impact force of the plasma pretreated specimen was 8.6% and 7.8% higher, respectively. Under the impact energy of 60 J, the peak impact force of the plasma pretreated specimen was 24.8% higher due to the mixed failure modes.

### 3.3. Damage Assessment

**Figure 10:**
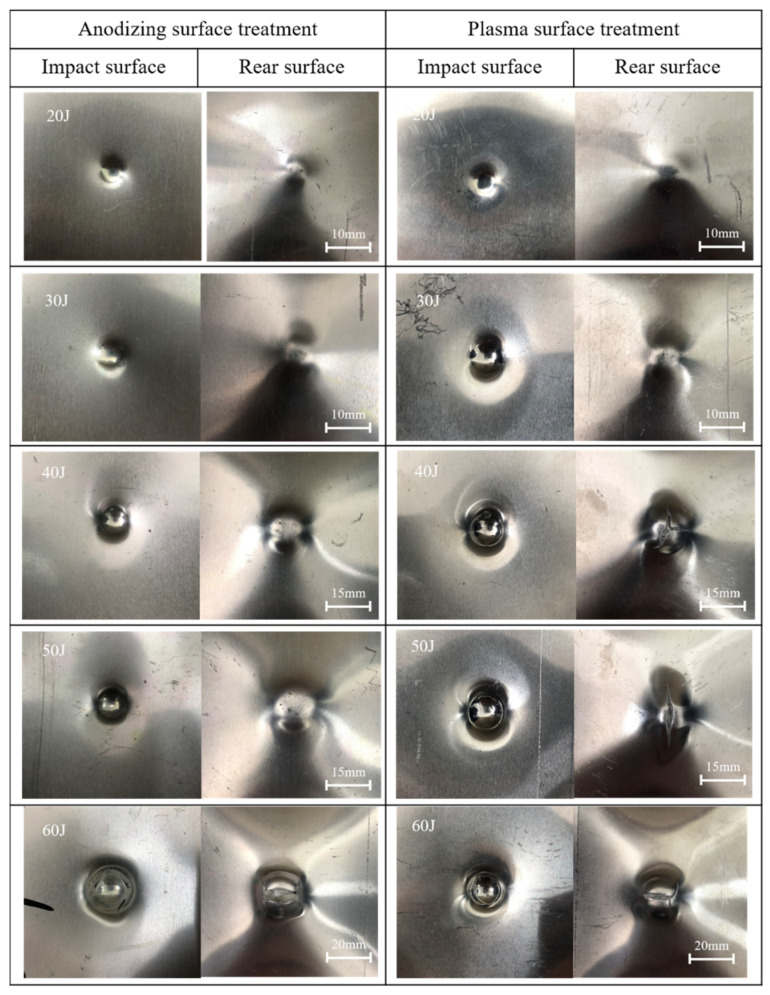
Low magnification optical micrographs of impacted Al/Gf/PP laminates with different surface treatments on the aluminum alloy sheet.

**Figure 11:**
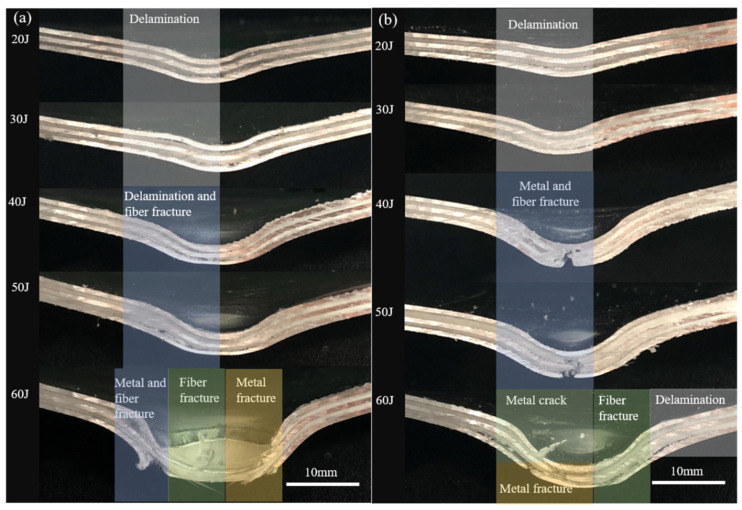
Close-up view of the cross-sections for two kinds of Al/Gf/PP laminates subjected to various low-velocity impact energy (20–60 J): (**a**) the aluminum alloy sheet modified by anodizing surface treatment, (**b**) the aluminum alloy sheet modified by plasma surface treatment.

Figure 10 and Figure 11 are the failure modes of the impact area and the cross-section of two kinds of Al/Gf/PP laminates under different low-velocity impact energy (20–60 J). When the impact energy was below 30 J, the visible damage mode that could be observed was the plastic deformation of the aluminum alloy sheet at the impact surface without any metal crack. Under the impact energy of 40 J and 50 J, the apparent difference was the outer-layer metal crack on the rear surface of plasma pretreated specimens. This is because the laminates after plasma pretreatment had a higher interlayer performance that possesses compressive forces generated at the manufacture time of cooling [42] and are more susceptible to stretching rather than delamination. Under the action of impact load, the aluminum alloy sheet was cracked without delamination failure. In addition, both types of laminates had a through-the-thickness failure at the low-velocity impact energy of 60 J.

According to the cross-sectional failure morphology of the impacted laminates in Figure 11, under the impact energy of 20 J and 30 J, the main failure mode of laminates was delamination, which is composed of elastic–plastic metal and linear elastic composite materials caused by different out-of-plane deformation trends during the rebound stage [43,44]. Currently, the influence of impact load was greater than the interlayer performance of the laminates [45]. Under the impact energy of 40 J and 50 J, fiber fracture can be seen in both laminates. The difference is that metal fracture occurred in the plasma pretreated specimen (Figure 11b). The overall bending of the laminate led to the fracture of the outer metal layer, and the local stress concentration led to the shear failure of the inner aluminum layer. The middle layer fractured due to the combination of global bending and local stress concentration.

**Figure 12:**
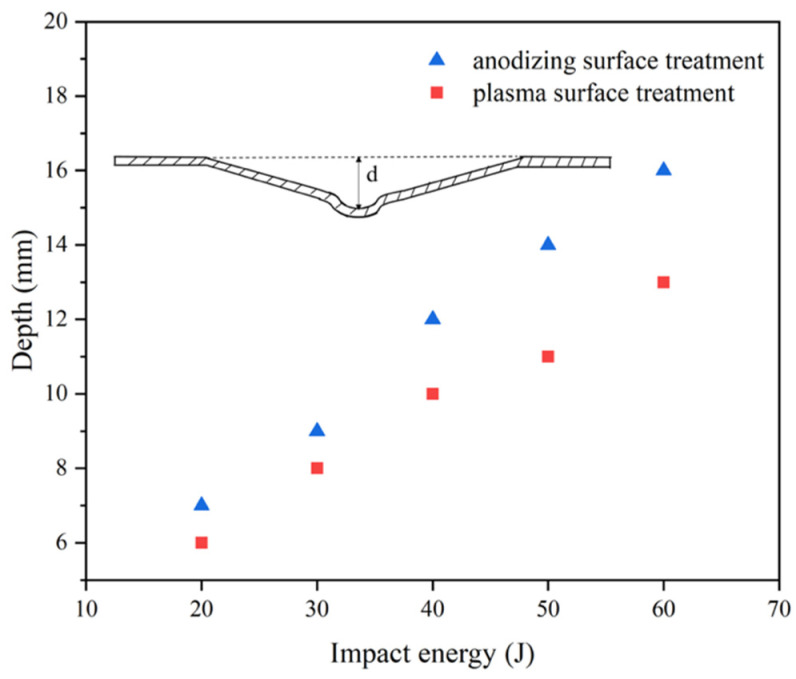
The influence of impact energy on the residual displacement of impacted Al/Gf/PP laminates with different surface treatments of the aluminum alloy sheet.

When two kinds of Al/Gf/PP laminates were subjected to the impact energy of 60 J, the fracture failure modes of the laminate were slightly different. In Figure 11a, the fracture failure of metal and fiber occurred on the rear surface of the laminate, and the glass fiber reinforced polypropylene layer could be observed on the impact surface. This was caused by the impact surface of the aluminum alloy sheet falling off during the cutting process, and the metal fracture area was large. From Figure 11b, all metal layers of the laminate were broken and the lack of interlayer fibers caused by fiber fracture failure further resulted in delamination failure. Figure 12 displays the residual displacement [46] of impacted Al/Gf/PP laminates with different surface treatments of aluminum alloy sheets. The increasing rate and values of residual displacement for laminates with modification of the plasma surface treatment were 14.3%, 11.1%, 16.7%, 14.8%, and 18.8% smaller than that by anodizing surface treatment under increasing impact energy. This suggests that larger bending deformation occurs due to the weak IPMC of anodizing pretreated specimens.

**Figure 13:**
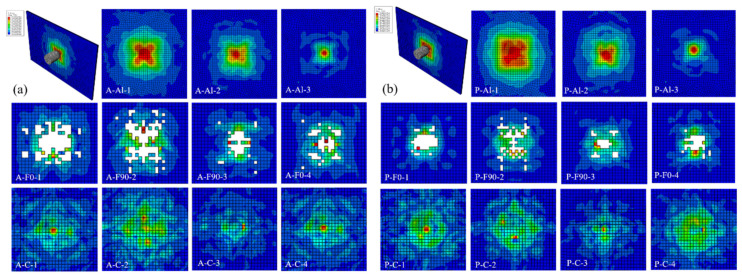
FEM simulation results of Al/Gf/PP laminates subjected to low-velocity impact energy of 20 J: (**a**) the aluminum alloy sheet modified by anodizing surface treatment, (**b**) the aluminum alloy sheet modified by plasma surface treatment.

Figure 13 displays the FEM simulation results of Al/Gf/PP laminates subjected to low-velocity impact energy of 20 J. For Al/Gf/PP laminates with different interlaminar shear properties, it was found that each layer (the aluminum alloy layer, the glass fiber reinforced polypropylene layer and the cohesive layer) exhibited different impact damage modes, particularly in the impact damage area of the glass fiber reinforced polypropylene layers. This indicates that the fiber layers were damaged before the aluminum alloy layers fractured since they play a role in transferring the impact force. The impact damaged area of the glass fiber layer in Figure 13b was smaller than that in Figure 13a, revealing the fact that the laminates with aluminum alloy sheets modified by plasma surface treatment possess better interlaminar shear properties. Table 5 summarizes the maximum von Mises stress of each layer in Figure 13. In the aluminum alloy layer by surface modification of anodizing and plasma, the maximum von Mises stress of the former in impact surface (Al-1) and middle layer (Al-2) was larger while the results of the rear surface (Al-3) were the same. Maximum von Mises stresses of the cohesive layer for laminates with the aluminum alloy sheet modified by plasma surface treatment were larger than that by the anodizing surface treatment, especially in the layer of C-2 and C-3. This indicates that the cohesive layer can transfer more impact forces in laminates with the aluminum alloy sheet modified by plasma surface treatment, resulting in the smaller impact damage of the glass fiber reinforced polypropylene layer than that on the aluminum alloy layer. Moreover, the maximum von Mises stress of each fiber layer in laminates with the aluminum alloy sheet modified by plasma surface treatment were larger than that by the anodizing surface treatment, which illustrates that the better interlaminar shear properties of Al/Gf/PP laminates make it more effective in transferring and dispersing the impact force between the layers.

**Figure 14:**
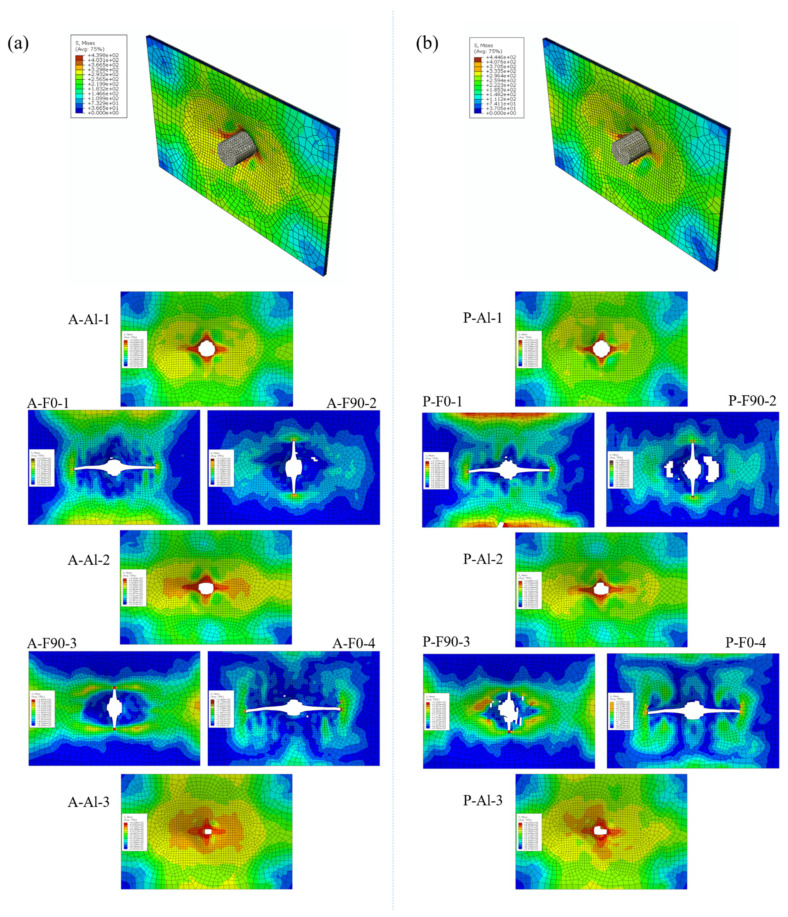
FEM simulation results of Al/Gf/PP laminates subjected to low-velocity impact energy of 60 J: (**a**) the aluminum alloy sheet modified by anodizing surface treatment, (**b**) the aluminum alloy sheet modified by plasma surface treatment.

Figure 14 displays the FEM simulation results of Al/Gf/PP laminates subjected to low-velocity impact energy of 60 J. Consistent with the experimental results, the specimens all suffered penetration failure under the impact of this energy level. However, the units with a larger damage value than the set damage value in the aluminum alloy (Al-1, Al-2, and Al-3) and composite material (F0-1, F90-2, F90-3, and F0-4) layers had been deleted, which were different from the actual results. The neglect of the thermal residual stress [47] in the FEM models could be responsible for this phenomenon. In the subsequent simulation process, thermal residual stresses could be considered in FEM models in the form of a predefined field. Damaged cracks of the composite material layer extend along the continuous fiber direction, and the damaged cracks of the aluminum alloy layer extend along the rolling direction of the metal. In the fiber layer, the stress perpendicular to the crack propagation direction was larger, especially at the edge of the specimen. In the metal layer, the stress expanded from the impact area to the surroundings and gradually weakened. The maximum von Mises stress and damaged area of the aluminum alloy layer after plasma pretreatment under the impact energy of 60 J was slightly larger than that of the anodized specimen. The interface also plays a role in stress transmission. Specimens with better interface properties enable the laminates to absorb more energy, which can be reflected in the energy absorption analysis below.

### 3.4. Energy Absorption

**Figure 15:**
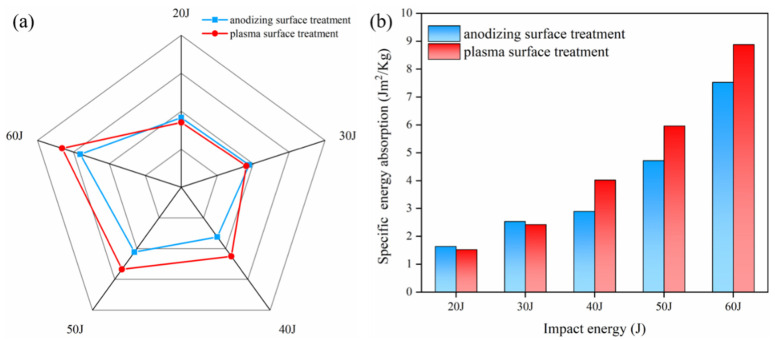
Al/Gf/PP laminates with different surface treatments of the aluminum alloy sheet in the process of low-velocity impact experiments: (**a**) energy absorption, (**b**) specific energy absorption.

The energy absorption (EA) is determined by calculating the area under the force–displacement trajectory and the result is displayed in Figure 15a. Under the impact energy of 20 J and 30 J, the EA of the anodized pretreated specimens was 7.4% and 4.5% higher than that of the plasma pretreated specimens, respectively. The effect of different IPMC can be regarded as similar within the allowable range of error. The EA of plasma pretreatment laminates with 40 J, 50 J, and 60 J impact energy were 39%, 27%, and 18% higher, respectively, and the energy absorption rate was higher than 50%. This is because the superior IPMC allows for more energy to be absorbed by the fracture of the metal and fiber before delamination occurs. Figure 15b shows the specific energy absorption (SEA) of laminates with different surface treatments. The surface density of the Al/Gf/PP laminates was 5.6 kg/m^2^. As the impact energy increased, the comparison results in SEA were consistent with that of EA. The maximum SEA was close to 9 Jm^2^/kg when the laminates with the modification of plasma surface treatment were subjected to the impact energy of 60 J. Zhou et al. [48] investigated four configurations of FMLs based on glass fiber/epoxy prepreg materials and three types (7075-O, 6061-O, 6061-T6) of aluminum alloy pretreated by an etching process. The results showed that the maximum SEA was below 8 Jm^2^/kg with the laminates using a six-series aluminum alloy. This indicates that the surface treatment of anodizing used in our study reached the industry level. Moreover, the plasma surface treatment investigated for manufacturing TP-based FMLs was superior to other conventional methods.

## 4. Conclusions

Based on the research in this paper, the following conclusions can be drawn:

1. From the result of the mode I interlaminar fracture toughness of Al/Gf/PP laminates, the fibers in the GFPP layer played a role in the fiber bridging effect at the interface between the metal and the composite material.
2. The increasing rate and values of residual displacement for laminates with modification of plasma surface treatment were smaller than that by anodizing surface treatment under increasing impact energy. Larger bending deformation occurred due to the weak IPMC of anodizing pretreated specimens.
3. Under low-velocity impact energy of 20 J and 30 J, the main failure mode of the laminates was delamination. Combined with the FEM simulation results, the fiber layers had been damaged and played a role in transferring the impact force.
4. When the impact energy was between 40 J and 50 J, there were metal cracks on the rear surface of plasma pretreated specimens, which possessed higher energy absorption and impact resistance although the integrity of the laminates could not be preserved. Due to the residual compressive stress generated during the cooling process, the laminates were more susceptible to stretching rather than delamination.
5. For impact energy (60 J) causing the through-the-thickness crack of plasma pretreated specimens, it exhibited higher SEA values close to 9 Jm^2^/kg due to better IPMC. From the FEM simulation results, the maximum von Mises stress and damaged area of the aluminum alloy layer after plasma pretreatment was slightly larger than that of the anodized specimen. The interface played a role in stress transmission and specimens with better interface properties enabled the laminates to absorb more energy.

## Figures and Tables

**Table 1:** The basic performance of the GFPP prepregs.

Fiber Orientation (°)	Fiber Content (% WT)	Density (g/cm^3^)	Thickness (mm)	Tape Width (mm)	Tensile Strength (MPa)	Flexural Strength (MPa)
0	60	1.38	0.30	1300	520	430

**Table 2:** Material properties of the aluminum alloy sheet (6061-T6) according to Johnson–Cook plasticity model.

Parameters	Values
Elastic	*E* = 68.90 GPa, *μ* = 0.33
Plastic	*A* = 324 MPa, *B* = 114 MPa, *n* = 0.42, *m* = 1.34
Damage	*d*_1_ = −0.77, *d*_2_ = 1.45, *d*_3_ = −0.47, *d*_4_ = 0

**Table 3:** Mechanical properties of the glass fiber reinforced polypropylene layer.

Mechanical Constant	Value
Young’s modulus in 1-direction, *E*_1_	30,700 MPa
Young’s modulus in 2-direction, *E*_2_	4800 MPa
Young’s modulus in 3-direction, *E*_3_	4800 MPa
Poisson’s ratio, *μ*_12_	0.16
Poisson’s ratio, *μ*_13_	0.16
Poisson’s ratio, *μ*_23_	0.16
Shear modulus, *G*_12_	2900 MPa
Shear modulus, *G*_13_	2900 MPa
Shear modulus, *G*_23_	2900 MPa
Beta damping parameter	1 × 10^−9^
Ultimate tens stress in 1-direction, *X*_1*T*_	400 MPa
Ultimate comp stress in 1-direction, *X*_1*C*_	235 MPa
Ultimate tens stress in 2-direction, *X*_2*T*_	13 MPa
Ultimate comp stress in 2-direction, *X*_2*C*_	30 MPa
Ultimate tens stress in 3-direction, *X*_3*T*_	13 MPa
Ultimate comp stress in 3-direction, *X*_3*C*_	30 MPa
Ultimate shear stress, *S*_12_	56 MPa
Ultimate shear stress, *S*_13_	56 MPa
Ultimate shear stress, *S*_23_	56 MPa

**Table 4:** Parameters of IPMC of the Al/Gf/PP laminates by different surface treatments.

Parameters of IPMC		Plasma	Anodizing
Density (kg/m^3^)	*ρ*	900	
Initial stiffness (N/mm)	$K_{nn}^{0}=K_{SS}^{0}=K_{tt}^{0}$	1.32 × 10^6^	
Interfacial strength (MPa)	$t_{n}^{0}$	3.980	1.640
	$t_{s}^{0}=t_{t}^{0}$	40.0	23.8
Fracture energy (kJ/m^2^)	$G_{IC}$	0.440	0.350
	$G_{IIC}=G_{IIIC}$	0.500	0.247
Mixed mode index	*η*	0.63 [39]	

**Table 5:** Maximum von Mises stress of each layer in Figure 13. (S/MPa).

Surface Treatment	Al-1	Al-2	Al-3	F0-1	F90-2	F90-3	F0-4	C-1	C-2	C-3	C-4
Anodizing (A)	359.1	355	340.3	126.8	107.8	112	86.1	74.6	52.3	82.8	44.8
Plasma (P)	356.6	348.2	340.8	226.7	144	135.6	106.9	74.9	82.8	101.2	48.1
